# Diversifying selection signatures among divergently selected subpopulations of Polish Red cattle

**DOI:** 10.1007/s13353-019-00484-0

**Published:** 2019-01-26

**Authors:** Artur Gurgul, I. Jasielczuk, E. Semik-Gurgul, T. Szmatoła, A. Majewska, E. Sosin-Bzducha, M. Bugno-Poniewierska

**Affiliations:** 1 Department of Animal Molecular Biology, National Research Institute of Animal Production, Krakowska 1, 32-083 Balice, Poland; 2 Department of Cattle Breeding, National Research Institute of Animal Production, Krakowska 1, 32-083 Balice, Poland; 3 Department of Nutrition Physiology, National Research Institute of Animal Production, Krakowska 1, 32-083 Balice, Poland; 4 University of Agriculture in Krakow, Institute of Veterinary Sciences, Mickiewicza 24/28, 30-059 Kraków, Poland

**Keywords:** β-Defensins, Milk production, Polish Red cattle, Selection signatures

## Abstract

Polish Red cattle is one of the few indigenous breeds of European red cattle which is characterized by several desired features, such as high disease resistance, good health, longevity, good fertility, and high nutritional value of milk. Currently, Polish Red cattle population is a subject of two independent breeding programs: (i) improvement program and (ii) genetic resources conservation program. The aim of the improvement program is the genetic progress in terms of milk production and body conformation traits, while the conservation program mainly focuses on protection of the genetic resources of Polish Red cattle and preservation of the existing, original gene pool. By the analysis of *F*_ST_ genetic distances across genome-wide SNP panel, we detected diversifying selection signatures among these two subpopulations and indicated (among others) the significance of *DGAT1* and *FGF2* genes for milk production traits in these cattle. We also found that among genes being presumably under selection in terms of milk production, there are genes responsible, for example, for mammary gland development (e.g., *SOSTDC1*, *PYGO2*, *MED1*, and *CCND1*) and immune system response (e.g., *IL10RA*, *IL12B*, and *IL21*). The most important finding of this study is that the most pronounced genetic differences between the analyzed populations were associated with β-defensin genes (e.g., *DEFB1*, *DEFB4A*, *DEFB5*, *DEFB7*, *DEFB10*, *DEFB13*, *EBD*, *BNBD-6*, and *LAP*) located within so-called bovine cluster D on BTA27. The β-defensins are expressed mainly in the mammary gland and are antimicrobial peptides against the Gram-negative and Gram-positive bacteria, viruses, and other unicellular parasites. This suggests that antimicrobial resistance of mammary gland is of high importance during selection towards increased milk production and that genes responsible for this process are selected together with increasing levels of productivity.

**Electronic supplementary material:**

The online version of this article (10.1007/s13353-019-00484-0) contains supplementary material, which is available to authorized users.

## Introduction

Polish Red cattle is one of the few indigenous breeds of European red cattle. It is characterized by several features typical for primitive populations, such as high disease resistance, good health, longevity, very good fertility, easy births, ease of calf rearing, and high biological value of milk. The Polish Red cattle is also characterized by a good adaptation to harsh environmental conditions, which is especially visible in the ability to limit the efficiency (enabling survival of seasonal feed deficiencies), as well as the relatively quick regeneration after condition loss. These features make the cattle of this breed well adapted to mountainous and submountainous living and production conditions (Szarek et al. 2004).

Currently, Polish Red cattle population is a subject of two independent breeding programs: (i) improvement program and (ii) genetic resources conservation program (Adamczyk et al. 2008). The aim of the improvement program is the genetic progress in terms of milk production and body conformation traits, leading to the refinement of the economic aspects of breeding and preservation of the existing beneficial functional features. Specialization is directed at the characteristics that have a fundamental impact on improving the profitability of milk production, like milk yield, protein yield, fat yield, and functional features with special emphasis on the udder morphology and leg health. The aim of the conservation program is to protect the genetic resources of Polish Red cattle used for both meat and dairy production, to preserve the existing, original gene pool, genetic variability of the population, and to maintain the productivity of Polish Red cattle at an acceptable level. Maintaining the largely primitive character of this breed is one of the major breeding goals for this part of the population (Adamczyk et al. 2008).

Despite both these subpopulations originate from the same local red cattle population (Szarek et al. 2004), the application of the two different selection programs could have generated genetic variability among the studied groups. Therefore, in this study, we attempt to identify diversifying selection signatures among these two Polish Red cattle populations. The obtained results presumably should allow us to identify genome regions with differentially selected variants which are responsible for milk traits and functional features selected in the analyzed populations. To this end, we used the well-established *F*_ST_-based approach which measures the genetic differentiation due to locus-specific allele frequencies variation between populations. With this approach, we identified numerous candidate genes for traits selected in both populations with especially strong reference to the udder health and udder developmental processes.

## Material and methods

The study material comprised 60 samples of ear tissue collected from cows belonging to Polish Red breed and included in “conservation” (RP; *n* = 37) and “improvement” (RE; *n* = 23) breeding programs. The animals were randomly selected and verified to be unrelated for at least two generations. The animals were coming from at least three different herds. All animal procedures were approved by the Local Animal Care Ethics Committee No. II in Kraków—permission number 1293/2016 in accordance with EU regulations. The genomic DNA was purified using Sherlock AX kit (A&A Biotechnology, Poland) and after quality control was genotyped with the use of BovineSNP50v2 BeadChip assay (Illumina, San Diego, CA) according to the standard Infinium Ultra protocol. Only samples with call rate > 0.95 were retained for analysis. The initial markers set included 54,609 SNPs and were further filtered to remove markers without known chromosomal position or located on sex chromosomes. SNPs that had minor allele frequency (MAF) < 0.05, genotyping rate < 0.8 (in joint populations), and deviated from Hardy-Weinberg equilibrium with *p* < 0.0001 (in each population separately) were also removed. This filtering step allowed retaining for further analysis a common set of 43,165 SNPs with average inter-marker distance of 57.9 kb in UMD3.1 genome assembly.

The across-genome breeds genetic differentiation was evaluated using pairwise *F*_ST_ distances (Weir and Cockerham 1984) measuring locus-specific allele frequencies variation between populations. *F*_ST_ values obtained using Plink software were further averaged in 10-SNP sliding windows to account for stochasticity in locus-by-locus variation. The window-averaged *F*_ST_ values were then ranked and top 1% of the observations pointed to windows with the most pronounced selection signals. Overlapping windows with the top *F*_ST_ values were subsequently merged and genomic regions spanned by this merged widows were extended on both ends by 25 kb to account for extended linkage. The resulting genome regions spanning the strongest diversifying selection signals were finally analyzed in details to identify encoded genes and their associated biological processes using UCSC Genome Browser (Karolchik et al. 2004), ENSEMBL database, and KOBAS Web Server (Xie et al. 2011). The gene overrepresentation tests (in GO categories) were performed according to all annotated bovine genes with correction for multiple testing.

The linkage disequilibrium (LD) and haplotype block structure at the most divergently selected regions among the studied populations were analyzed using HaploView 4.2 (Barrett et al. 2005) software examining pairwise LD on the distance up to 500 kb and detecting blocks based on a method proposed by Gabriel et al. (2002).

Global population differentiation was analyzed using principal component analysis (PCA) based on individual genotypes and mean or weighted *F*_ST_ distances.

### Data availability

The datasets used and/or analyzed during the current study are available from the corresponding author on reasonable request.

## Results

**Fig. 1:**
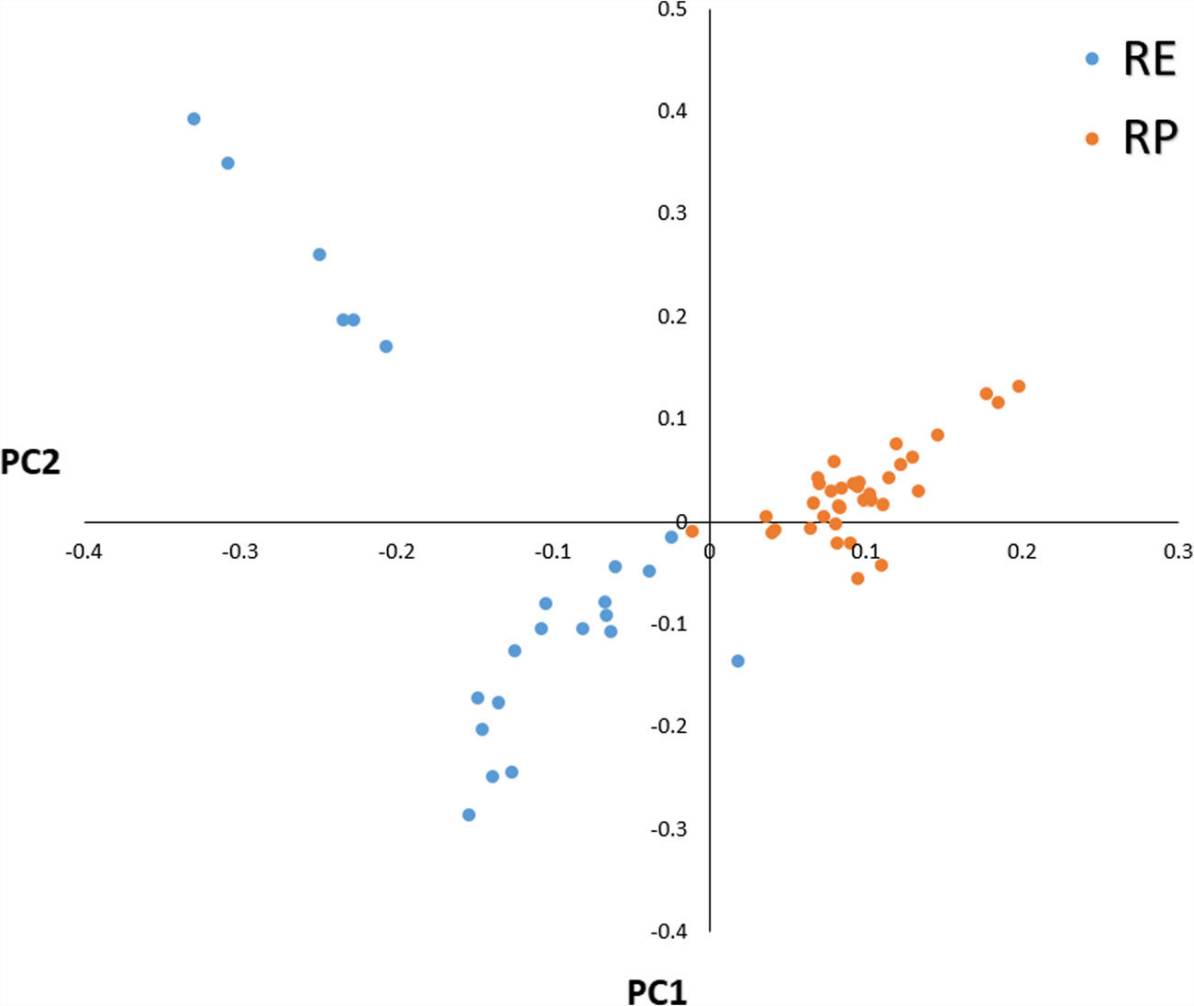
Principal component analysis for the studied individuals. *RP*, conserved population; *RE*, improved population

**Fig. 2:**
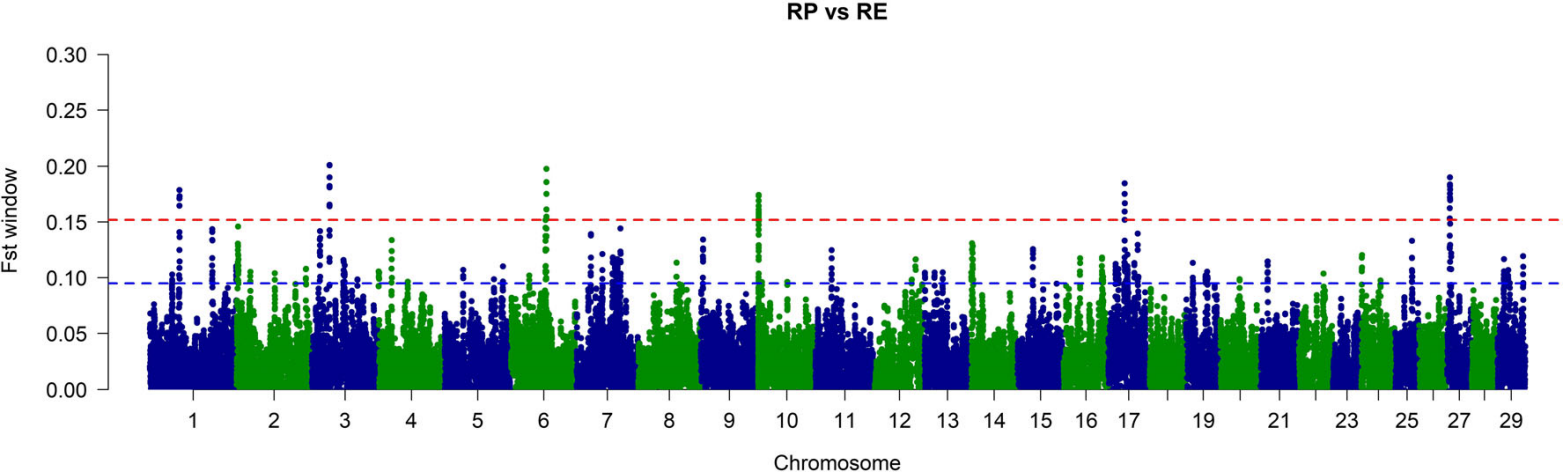
Plot of window-averaged *F*_ST_ values across all bovine autosomes. The 10 SNP sliding window–averaged *F*_ST_ values are plotted against centered genomic positions of windows. *Blue dashed line* shows threshold of 1% of the top *F*_ST_ observations. *Red dashed line* represents 0.1% of the top *F*_ST_ observations. *RP*, conserved population; *RE*, improved population

The used SNP panel showed sufficient polymorphism parameters in both populations with average MAF in RP of 0.275 and 0.272 in RE. The mean observed heterozygosity was comparable for both populations and was 0.369 for RP and 0.377 for RE (Table 1). The general population differentiation was rather low with mean and weighted *F*_ST_ distances of 0.0226 and 0.0242, respectively. The PCA showed, however, that both populations form clearly separated clusters of observations with visibly higher level of genetic variation observed in RE population (Fig. 1). RE population was additionally subdivided into two visible clusters. A local genetic differentiation of the populations was predominantly concentrated in 76 separate genomic regions located on 25 different autosomes (Supplementary File 1). No strong selection signals were identified on chromosomes 18, 23, 26, and 28. The most pronounced differential selection signals were observed on BTA1, BTA3, BTA6, BTA10, BTA17, and BTA27 (Fig. 2, Table 2). The genome regions overlapped by the detected selection signals spanned from 26.4 kb to 4.6 Mb and encompassed 610 different genes (Supplementary Files 1 and 2). The analysis of biological processes associated with the genes allowed for detection of very general GO categories including primary metabolic process (143 genes), regulation of protein phosphorylation (34 genes), regulation of protein kinase activity (21 genes), phosphorylation (46 genes), growth factor activity (8 genes), mammary gland development (5 genes), and immune system processes (28 genes). Among the detected genes, we also identified previously described candidate genes for milk traits like *DGAT1* and *FGF2* (Ogorevc et al. 2009) or disease resistance-associated genes, like *IL10RA*, *IL12B*, and *IL21* (Malkovský et al. 1988). The detailed analysis of the genome regions spanned by the selection signatures with the top 0.1% of the *F*_ST_ values (strongest diversifying selection signals) allowed for detection of 46 overlapped genes. The overrepresentation test performed for those genes showed single enriched GO category connected with defense response to bacterium (adj*P* = 0.02). The genes associated with this process belonged mainly to the β-defensins family (e.g., *DEFB1*, *DEFB4A*, *DEFB5*, *DEFB7*, *DEFB10*, *DEFB13*, *EBD*, *BNBD-6*, *LOC783012*, *LAP*). In the analyzed genes set, we also detected other immune system functioning–related genes, like *CD53* and *TRAT1* (Zhang and Samelson 2000; Todros-Dawda et al. 2014).

**Table 1:** Marker statistics and basic population indexes

Population	*n*	SNPs (*n*)	Mean SNPs distance (kb)	Mean MAF	Ho	He	*F*_ST_ (mean/weighted)	*F*
RP	37	43,165	57.97	0.275	0.369	0.363	0.0226/0.0242	− 0.018
RE	23			0.272	0.377	0.359		− 0.049

*RP*, cows under “conservation program”; *RE*, cows under “improvement program”; *Ho*, observed heterozygosity; *He*, expected heterozygosity; *MAF*, minor allele frequency; *F*, inbreeding coefficient based on the observed versus expected number of homozygous genotypes

**Table 2:** Genomic regions spanned by the strongest detected selection signals (with top 0.1% of *F*_ST_ values)

Chr	Start	End	Size	Genes*
1	54,052,439	54,625,163	572,724	*TRAT1—T Cell Receptor Associated Transmembrane Adaptor 1*
				*MORC1—MORC Family CW-Type Zinc Finger 1*
				*DPPA2—Developmental Pluripotency Associated 2*
3	32,527,488	32,883,278	355,790	*LRIF1—Ligand-Dependent Nuclear Receptor Interacting Factor 1*
				*CD53—Cell Surface Glycoprotein CD53*
				*KCNA3—Potassium Voltage-Gated Channel Subfamily A Member 3*
6	63,018,446	63,186,617	168,171	*ATP8A1—ATPase Phospholipid Transporting 8A1*
6	64,418,457	64,662,856	244,399	–
10	766,191	832,105	65,914	*MCC - MCC*, *WNT Signaling Pathway Regulator*
17	29,760,863	30,508,078	747,215	*LOC101903064—Uncharacterized protein*
				*PGRMC2—Progesterone Receptor Membrane Component 2*
				*LARP1B—La Ribonucleoprotein Domain Family Member 1B*
				*ABHD18—Abhydrolase Domain Containing 18*
				*MFSD8—Major Facilitator Superfamily Domain Containing 8*
				*PLK4—Polo Like Kinase 4*
				*HSPA4L—Heat Shock Protein Family A (Hsp70) Member 4 Like*
				*SLC25A31—*S*olute Carrier Family 25 Member 31*
				*INTU—Inturned Planar Cell Polarity Protein*
27	4,947,815	6,243,961	1,296,146	*SPAG11—Sperm-associated Antigen 11*
				*ZNF705A—Zinc Finger Protein 705A*
				*LAP—Laryngeal Adductor Paralysis*
				*DEFB7—Beta-defensin 7*
				*BNBD-6—Dfensin Beta 6*
				*DEFB4A—Defensin Beta 4A*
				*DEFB—Defensin*, *Beta*
				*DEFB13—Defensin Beta 113*
				*EBD—Enteric Beta-defensin*
				*DEFB5—Defensin*, *Beta 5*
				*DEFB10—Defensin*, *Beta 10*
				*DEFB1—Defensin*, *Beta 1*
				*GPM6A—Glycoprotein M6A*

*Genes with no assigned name or pseudogenes were not included in the table

**Fig. 3:**
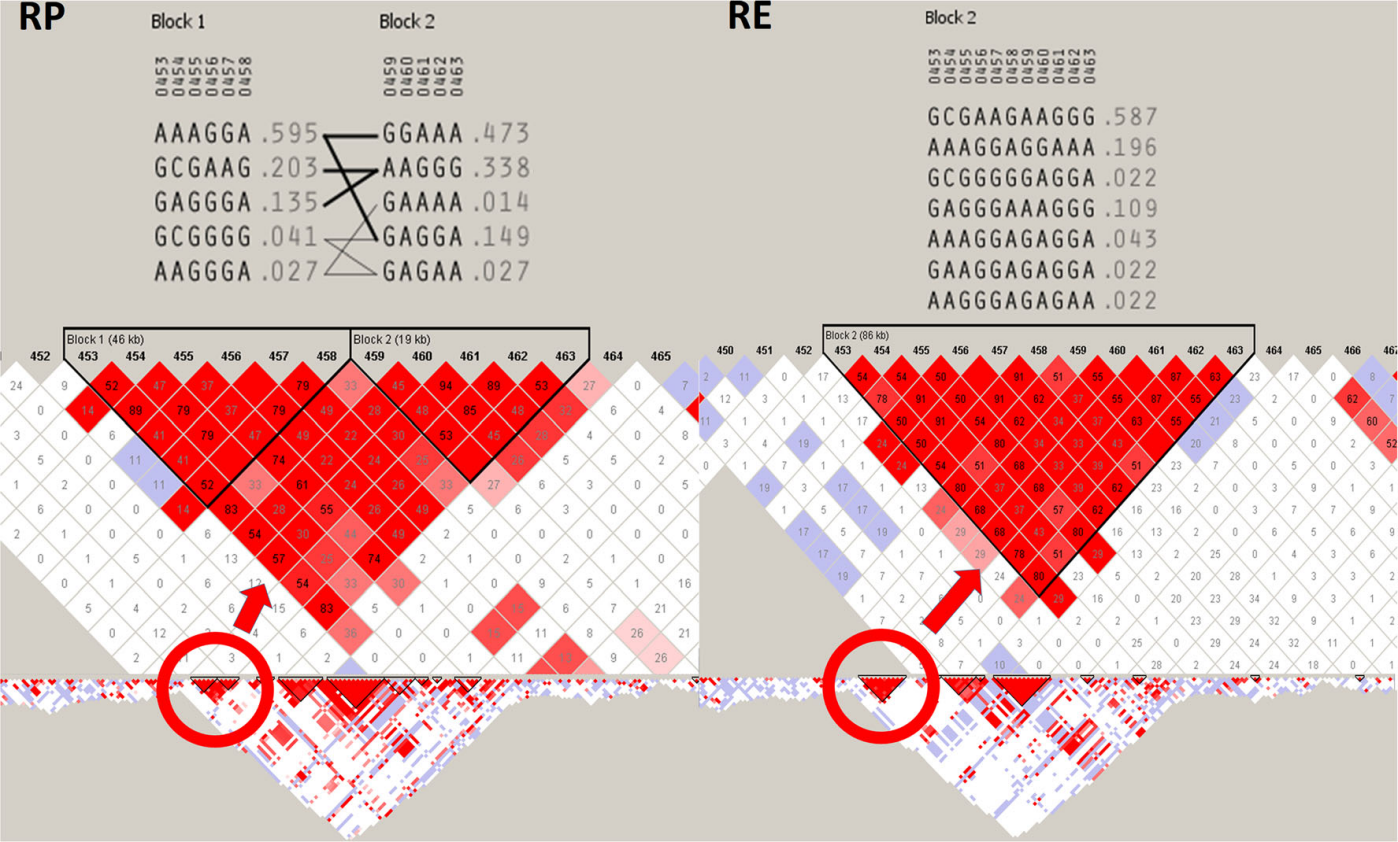
Linkage disequilibrium and haplotype block structure at the strongest detected diversifying selection signal on BTA3 between 32.5 and 32.9 Mb of genomic sequence. RP, population under “conservation program”; *RE*, population under “improvement program”. LD nodes were colored according to *D*′ values and *R*^2^ values were plotted inside the nodes. *Red circle* marks the haplotype blocks spanned by the detected selection signal. Haplotypes sequences and frequencies are presented on the top

Two selection signals found in this study included, inter alia, the strongest detected signal on BTA3 (32.5–32.9 Mb) and the signal spanning the largest genomic region (1.3 Mb) among the signals with top 0.1% of *F*_ST_ values (BTA27; 4.9–6.2 Mb). These two selection signals were analyzed in details to identify their possible meaning and evaluate haplotype structure at these loci. The analysis of the divergently selected region on BTA3 showed that the detected selection signal colocalized with the large chromosomal region of strong LD (31.6–33.5 Mb) and encompassed large haplotype block with high-frequency haplotypes in both studied populations (> 0.5). The detailed analysis of haplotypes showed that in separate populations, the most common haplotypes were composed of alternative SNP variants. Additionally, only haplotypes found in RP breed showed some minor signs of recombination (Fig. 3). The analyzed region on BTA3 overlapped with four genes (*LRIF1—Ligand-Dependent Nuclear Receptor Interacting Factor 1*, *ENSBTAG00000042091*, *CD53—Cell Surface Glycoprotein CD53*, *KCNA*3—*Potassium Voltage-Gated Channel Subfamily A Member 3*) including one non-coding small nuclear RNA.

**Fig. 4:**
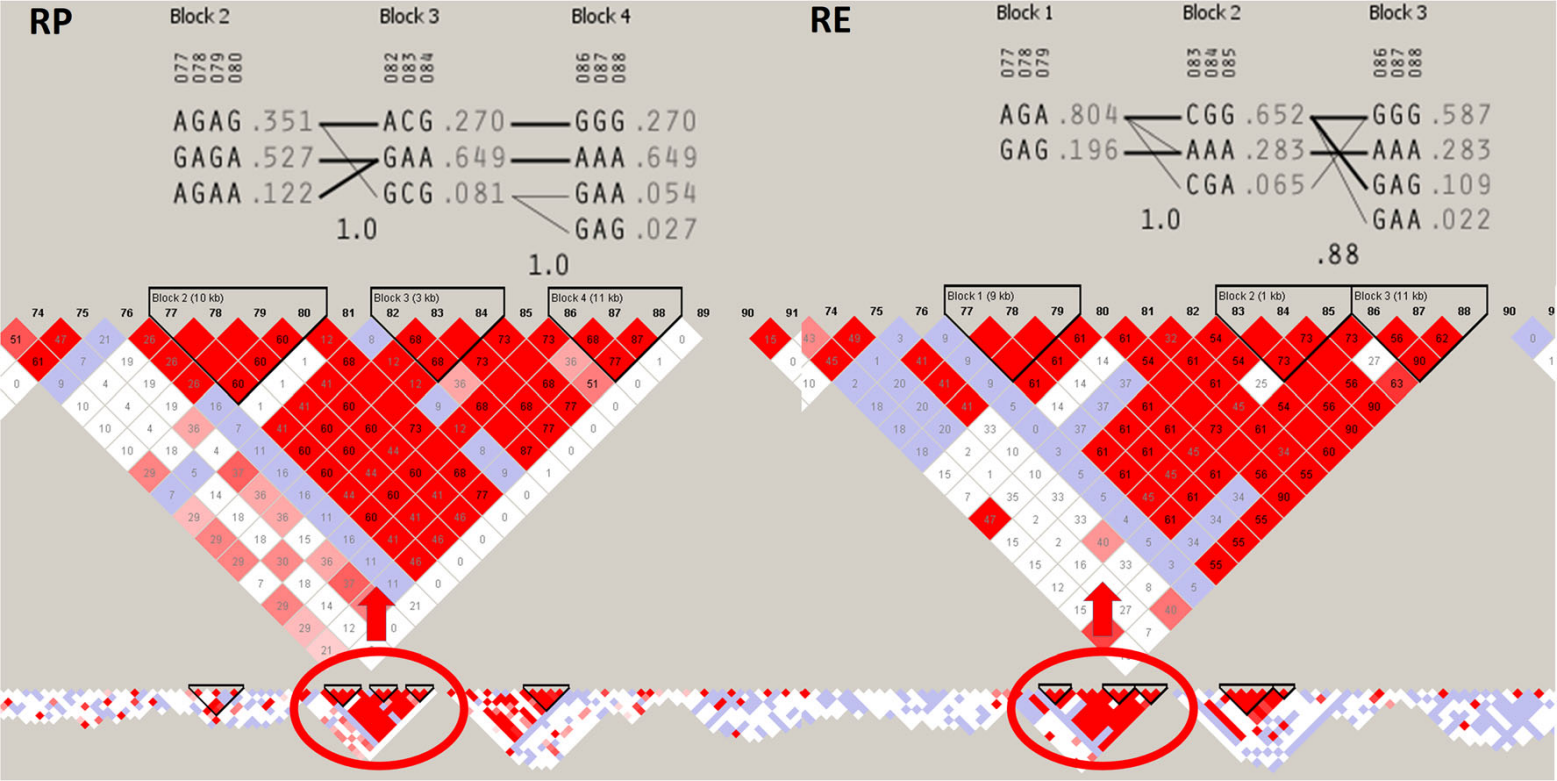
Linkage disequilibrium and haplotype block structure at the largest strong diversifying selection signal on BTA27 between 4.9 and 6.2 Mb of genomic sequence. *RP*, conserved population; *RE*, improved population. LD nodes were colored according to *D*′ values and *R*^2^ values were plotted inside the nodes. *Red circle* marks the haplotype blocks spanned by the detected selection signal. Haplotypes sequences and frequencies are presented on the top

The genomic region on BTA27 overlapping with the detected large and strong diversifying selection signal was also characterized by strong LD (unique on the chromosome scale) and encompassed three haplotype blocks showing common variants with haplotype frequency above 0.5. The detailed analysis of haplotypes again showed signs of diversifying selection among the analyzed populations, represented by haplotype variation and divergent selection of separate variants (Fig. 4). The signal on chromosome 27 overlapped with 21 different genes, mainly belonging to β-defensins family (Table 2) grouped in bovine cluster D (Gurao et al. 2017).

## Discussion

**Fig. 5:**
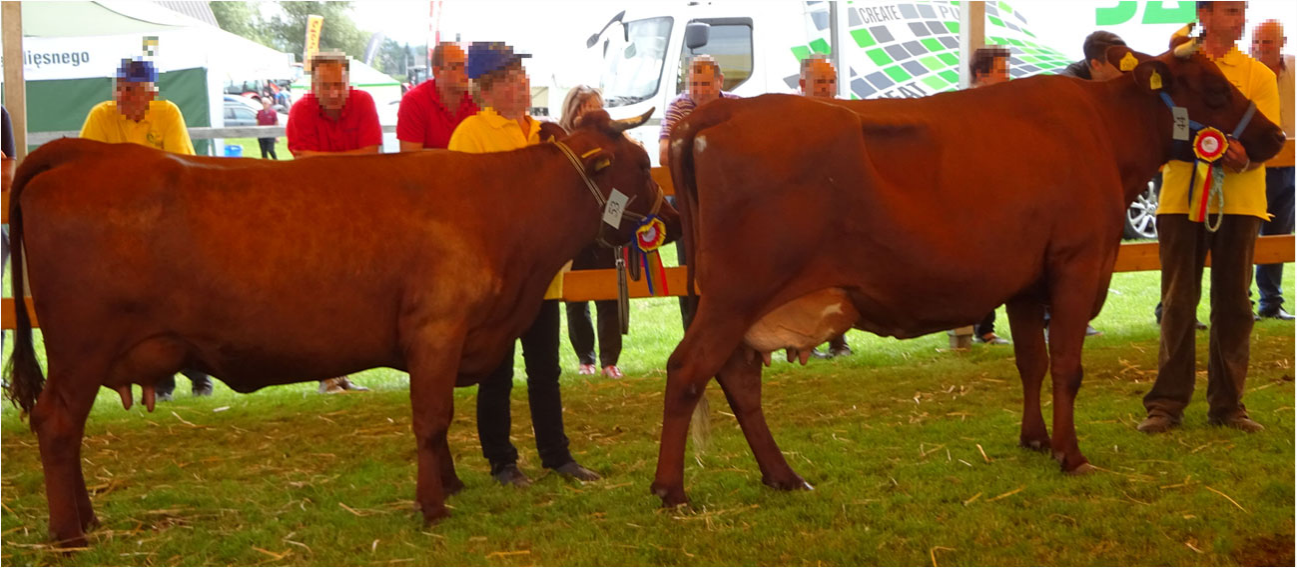
Two champion cows belonging to the “conservation program” (on the *left*; RP) and “improvement program” (on the *right*; RE). Clear differences in body constitution and udder morphology traits can be seen between conserved and improved animals (picture by Majewska A)

In this study, we attempt to identify diversifying selection signatures among two subpopulations of Polish Red cattle formed by animals subjected to conservation program (selected mainly towards primitive type and breed standard) and animals used for milk production (selected for milk and functional traits). These two populations visibly diversified over time and the phenotypical differences between them mainly refer to milk yield traits as well as exterior features which were strongly shifted towards milk production type in the improved cattle (Fig. 5).

The low average level of genetic differentiation among the studied populations (as shown by global polymorphism parameters) confirms their common origin from local population (Szarek et al. 2004) and suggests that major genetic differences between them are concentrated over the specific genomic *loci* which were divergently selected since the implementation of the improvement program. The performed PCA showed that the genomes of both these populations bear signs of differentiation with fraction of individuals being still genetically close. RE population was, however, subdivided into two visible clusters due to the different level of historical crossbreeding present in the pedigrees of the analyzed animals. The fact that both subpopulations originate from the same native population created an opportunity to trace allele frequency differences among them with high probability that they result from application of selection directed to improvement of milk traits in commercial population. This allowed for identification of candidate genes affecting presumably both primitive traits found in conserved population (being lost due to the intensive selection) (Lindhé and Philipsson 1998) but also *loci* of genes responsible for traits selected in the improved subpopulation.

The *F*_ST_-based approach applied in this study allowed us to identify several selection signals of which the most pronounced were localized on chromosomes 1, 3, 6, 10, 17, and 27. Among the genes associated with the detected signals, we found well-established fat yield and fertility markers like *DGAT1* (*Diacylglycerol O-Acyltransferase 1*) and *FGF2* (*Fibroblast Growth Factor 2*) (Ogorevc et al. 2009). Large portion of genes detected in this study was associated with immune system functioning or antimicrobial resistance and included several members of interleukins and β-defensins family. We also detected some genes responsible for mammary gland development (*SOSTDC1*, *PYGO2*, *MED1*, *CCND1*, *FGF2*), which may be of high importance for the improved population and its milking abilities (Gu et al. 2009; Närhi et al. 2012; Casimiro et al. 2013; Marete et al. 2018). A revision of the gene functions showed that *SOSTDC1* (*Sclerostin Domain Containing 1*) controls the size and shape of mammary buds in mice (Närhi et al. 2012), while *PYGO2* (*Pygopus Family PHD Finger 2*) ablation results in defective mammary morphogenesis and regeneration (Gu et al. 2009). Moreover, the *FGF2* gene (in addition to its effect on fat yield) was shown to play a role in development and reorganization of the mammary gland (Marete et al. 2018).

Within the selection signal on BTA19, we detected *LASP1* (*LIM and SH3 Protein 1*) gene. This gene was previously shown to have effect on body size regulation in horses (Makvandi-Nejad et al. 2012). It was also demonstrated that its expression level is 18-fold higher in lactating mammary tissue relative to non-lactating tissue in cows (Suchyta et al. 2003).

### The strongest detected selection signal on BTA3

The strongest detected diversifying selection signal found in this study was localized on BTA3 between 32.5 and 32.9 Mb of genomic sequence. This region was found to be closely positioned with previously described quantitative trait loci (QTL; 30,706,774–31,080,623 and 34,065,565–36,461,414) affecting milk yield, fat yield, and protein yield in Holstein and other cattle populations (Rodriguez-Zas et al. 2002; Boichard et al. 2003; Viitala et al. 2003; Ashwell et al. 2004; Ihara et al. 2004). In the study of Cohen-Zinder et al. (2011), genes such as *RAP1A*, *ADORA3*, and *C3H1orf88* were proposed as having a major effect on the milk traits within this QTL. The divergently selected region found in this study did not directly overlap with those genes; however, it was in a large region of high LD (unique in chromosome scale), spanning over 1.1 Mb (32.4–33.6 Mb) in both analyzed populations. Within the detected selection signal, we found only three genes, namely *LRIF1*, *CD53*, and *KCNA3*. These gene functions could be connected with both male fertility and immune system response. Exemplary, *LRIF1* product represses the ligand-induced transcriptional activity of retinoic acid receptor alpha (RARA) and disruption of retinoid signaling was shown to impair mammalian spermatogenesis and fertility (Chung et al. 2011). The region also displayed high marker linkage and extensive haplotype structure, representing possibly reduced genetic variation resulting from selection pressure directed on the analyzed locus.

### Extensive selection signal on BTA27

Among the strongest selection signals detected in this study (0.1% of highest *F*_ST_ values), the largest genomic region with signs of divergent selection was identified on BTA27 (1.29 Mb). This signal overlapped with several genes coding proteins belonging mainly to the β-defensins family (Table 2). β-Defensins are amphipathic cationic peptides that have been reported to function as antimicrobial peptides (AMPs) for the Gram-negative and Gram-positive bacteria, viruses, fungi, and other unicellular parasites (Brogden 2005). Their antimicrobial activity is mediated mainly through permeabilization of cell membrane (Huang 2000) or stimulation of hydrolases, therefore degradation of the cell wall (Bierbaum and Sahl 1985). The first β-defensin was isolated from bovine respiratory tract and was denoted as tracheal AMP (TAP—tracheal antimicrobial peptide) (Diamond et al. 1991). Currently, 58 β-defensin genes located within four clusters have been described in bovine genome on chromosomes 8, 13, 23, and 27 and were designated as cluster A, cluster B, cluster C, and cluster D, respectively (Meade et al. 2013). It was found that the genes important for resistance against intramammary infections are located on BTA27 in the cluster D (Gurao et al. 2017). These β-defensins are expressed both in the mammary gland and in the milk somatic cells, thus have a potential to prevent the intramammary infection (Gurao et al. 2017). In this study, among β-defensin genes overlapped by selection signal on BTA27, we detected, inter alia, *LAP*, *DEFB1*, *DEFB5*, *DEFB10*, and *DEFB4A*. Constitutive expression of *LAP* has been reported in the mammary gland of juvenile, lactating (both healthy and infected), and non-lactating cows (Roosen et al. 2004). Another study has shown that the *LAP* is expressed only in infected cattle (Swanson et al. 2004). The direct relationship between *LAP* and somatic cell count (SCC) has been also presented, where higher concentration of *LAP* in the milk of cattle infected with *Staphylococcus aureus*, *Streptococcus bovis*, *Streptococcus dysgalactiae*, and *E. coli* was observed than in uninfected cows (Kazuhiro et al. 2013). Similarly, *DEFB1* gene was found to be inducibly expressed during intramammary gland infection (Roosen et al. 2004), while *DEFB5* has been reported in mammary gland infected with coagulase-positive staphylococci and coagulase-negative staphylococci (Kościuczuk et al. 2014). Additionally, the expression of *DEFB10* was high in early lactation stages accompanied by infection by coagulase-positive staphylococci (Kościuczuk et al. 2014). What is important, β-defensins were also shown to have effect on milk traits, where β4-defensin (*DEFB4A*) polymorphisms were significantly associated with protein yield and fat or protein contents (Bagnicka et al. 2008) and milk yield (Krzyzewski et al. 2008) in Polish Holstein-Friesian cattle.

All these findings suggest that the selection signal detected on BTA27 in this study is associated with selection towards increased resistance against mastitis. Based on LD and haplotype block structure analysis, we presume that the observed allelic differences were created under the influence of relatively recent selection events. This is supported by the fact that genome regions being under strong ongoing selection are characterized by strong LD and extensive haplotype structure (Qanbari et al. 2014) while recombination mechanisms acting over several generations result in LD decay around variants selected a relatively long time ago in the population history (Slatkin 2008). Thus, we propose that both natural and artificial selection are mechanisms acting on BTA27 locus; however, in extensive and intensive breeding conditions, different microbial and environmental factors are of major importance for mammary health, and thus different variants are selected in the studied cattle populations. It is also a known fact that together with increased milk yield and milk synthesis intensity, the immune system activity is being altered. It was shown that selection towards increased milk yield has a detrimental effect on the health condition of the mammary gland (Rupp and Boichard 2003); thus, the mastitis is a problem mainly in high-production herds raised in intensive farming conditions.

## Conclusions

By the comparative analysis of divergently selected subpopulations of Polish Red cattle, we again indicated the importance of *DGAT1* and *FGF2* genes for milk production traits in cattle. We also found that among genes being under selection in terms of milk production in red cattle, there are genes responsible for mammary gland development such as *SOSTDC1*, *PYGO2*, *MED1*, and *CCND1* and immune system response like *IL10RA*, *IL12B*, and *IL21*. The most pronounced differences between the analyzed populations were, however, associated with β-defensin genes located within bovine cluster D on BTA27 which shows that antibacterial resistance of mammary gland is of high importance during selection towards increased milk production.

## Electronic supplementary material

ESM 1 Genomic regions formed by F_ST_ windows with the top 1% values. (XLSX 10 kb)

ESM 2 Genes found within top 1% of window-averaged F_ST_ values. (XLSX 61 kb)
